# Social Dynamics Are Associated with Plant-Based Dietary Consumption Patterns Among Rural Adults in the U.S.

**DOI:** 10.3390/nu18203346

**Published:** 2026-10-13

**Authors:** Sevda Alvirdizadeh, Saadatu Abdul-Rahaman, Rebecca A. Seguin-Fowler, Emily H. Belarmino

**Affiliations:** 1 Food Systems Program, University of Vermont, Burlington, VT 05405, USA; salvirdi@uvm.edu (S.A.); saadatu.abdul-rahaman@uvm.edu (S.A.-R.); 2 Gund Institute for Environment, University of Vermont, Burlington, VT 05405, USA; 3 Institute for Advancing Health Through Agriculture, Texas A&M AgriLife Research, College Station, TX 77843, USA; r.seguin-fowler@ag.tamu.edu; 4 Department of Nutrition, College of Agriculture and Life Sciences, Texas A&M University, College Station, TX 77843, USA; 5 Department of Nutrition and Food Sciences, University of Vermont, Burlington, VT 05405, USA

**Keywords:** plant-based diet, rural America, social norms, food systems

## Abstract

Background/Objectives: Social dynamics are known to influence eating behaviors and differ across geographic contexts. Some evidence suggests healthy plant-based diets (PBDs) are associated with reduced risk of chronic diseases, morbidity and mortality, and have lower environmental burdens. This paper examines the role of social influence, expectations, and pressures in consumption of PBDs among rural residents in the United States, with attention to gender differences. Methods: National survey data from rural adults (*n* = 2210) were analyzed using adjusted logistic regression models including an interaction between social beliefs and gender, and qualitative interviews (*n* = 55) thematically explored gendered social influences on PBD patterns. Results: Results showed that perceived expectations from important members of the respondents’ social network were positively associated with PBD adherence (*p* < 0.001), without a significant overall interaction with gender (*p* = 0.163). Perceived social pressure was also associated with PBD adherence overall (*p* = 0.009), and the association differed by gender (interaction *p* = 0.001), but did not show a consistent trend across levels of agreement. Qualitative findings suggested that family members influenced the feasibility of PBD, while friends were more commonly described as supportive. Women appeared to face challenges to enacting a PBD when family members (especially male partners) were not supportive. Conclusions: These results suggest social relationships have complex and gendered roles in shaping PBD among rural residents in the United States.

## 1. Introduction

In the United States, adults living in rural areas remain at increased risk for diet-related chronic diseases such as cardiovascular disease (CVD), diabetes, and cancer-leading causes of disability and death [1,2]. Improving diet quality among rural residents is a common target for efforts to improve rural health equity [3]. A 2023 systematic review found that plant-based diets (PBDs), which are characterized by limited consumption or elimination of animal-source foods are associated with lower risk of several chronic diseases, particularly when those PBDs followed a “healthy” profile, characterized by higher intake of vegetables, fruits, whole grains, and legumes [4]. Such diets may also generate fewer environmental burdens compared to standard diets [5]. Understanding the factors that influence healthy PBDs among rural residents, particularly in the context of overall diet quality, is essential to developing programs and policies that encourage their uptake in rural communities.

Research suggests that relationships and interpersonal dynamics can play an important role in shaping eating behaviors, even in rural areas [6]. Family members can influence dietary choices through encouragement, shared food, and expectations about acceptable meals [7]. Support may help people enact their preferences, whereas resistance to dietary change may create practical challenges [8]. Evidence remains limited on how rural adults experience such influences in relation to PBDs. These dynamics may play out differently for men and women given the presence of more traditional gender roles and identities in many rural communities and households [6]. Prior research has identified strong positive attitudes towards meat, high social expectations to consume meat, and daily meat consumption among rural residents [9]. These positive sentiments may be especially strong among men given traditional associations between meat and masculinity and the enduring stereotype that “real men eat meat” [10].

Although existing research provides support for social influences on rural dietary intake, these studies often focus on specific dietary components (e.g., fruit and vegetables) rather than total diet and infrequently consider the ways in which different spheres of influence (e.g., family, peers, community) shape eating behaviors. Evidence on PBD consumption from rural-specific contexts is limited. Further, there is a lack of evidence on how people of different genders experience social dynamics in relation to PBDs. The Theory of Planned Behavior (TPB) provides a framework for examining how attitudes, subjective norms (injunctive and descriptive), and perceived behavioral control relate to intentions and behavior. This study examines associations between PBD adherence and two forms of social norms among rural adults: perceived expectations from important others (injunctive norms) and perceived social pressure (descriptive norms), and explores household, family, friend, and community influences on PBDs.

## 2. Materials and Methods

This research leverages data from two complementary data sets: quantitative survey data with qualitative phenomenological inquiry. The research protocols were reviewed and determined exempt by the Institutional Review Board at the University of Vermont, STUDY00001425 approved 10 February 2021, STUDY00002156 approved 5 July 2022, STUDY00002636 approved 20 June 2023.

### 2.1. Quantitative Study

Data collection: A national rural online survey was conducted via Qualtrics in 2023 and 2024. A convenience sample was first recruited through paid advertisements on Facebook. To broaden racial diversity in the recruited sample, this was supplemented by direct email recruitment to self-identified Asian and Black rural adults on a marketing email list purchased from Exact Data. Eligible respondents (1) were 18 years or older; (2) had lived in the U.S. for at least five years, and (3) self-identified their residence as rural. Interested respondents were directed to the online survey for eligibility screening and consent. Respondents were eligible to participate in a drawing for a $99 gift card after completing the survey.

The survey asked about attitudes, social norms, perceived behavioral control, and other factors that influence PBDs as well as dietary intake and respondents’ personal characteristics. The present study used data from variables on social norms, PBD pattern adherence, and personal factors. Before answering survey questions, respondents were provided with a definition of a healthy plant-based dietary pattern as one emphasizing foods from plants while allowing varying levels of animal-product consumption. Respondents were asked: “Do you follow a plant-based diet?” (yes/no), and levels of agreement with two social norm statements adapted from Sparks et al. [11] were used to assess perceived social expectations and social pressure regarding PBD (Table 1).

Data analysis: Of the 2786 survey responses, 2210 participants were included in either of the analyses. Missing data in the logistic regression analyses were handled using model-specific complete-case analysis. Consequently, the model examining perceived expectations from important others included 2209 respondents, and the model examining perceived social pressure included 2186 respondents. The same approach was applied separately to the interaction analyses. Among the valid cases, descriptive statistics were used to summarize each variable, and frequencies were compared between respondents who did and did not report adherence to a PBD using chi-square tests with Bonferroni-adjustments for pairwise comparisons. We used binary logistic regression to examine associations between social norm measures and PBD adherence. Two separate models were estimated: one included perceived social pressure and the other included perceived expectations from important others. Each five-point agreement item was entered as a categorical predictor using indicator coding, with “Strongly disagree” as the reference. Gender, age, education, and income were included as covariates in both models. Separate interaction models included gender interactions with social norm measures, and overall Wald tests assessed whether associations differed by gender. Pairwise comparisons on the logit scale compared the five response categories in models without interactions and the ten combinations of gender and response category in models with interactions. Šidák adjustments were applied within each set of comparisons to account for multiple testing. All statistical analyses were performed using the Statistical Package for Social Science (version 20; IBM Corp., Armonk, NY, USA), with *p*-values < 0.05 being considered significant.

### 2.2. Qualitative Study

Participants: Rural residents from Vermont and Texas were recruited for semi-structured interviews using newspaper advertisements, digital postings on social media, physical posts in restaurants and food retailers, and snowball sampling. The first set of interviews (*n* = 27), conducted between July and December 2023, focused on the general population. Inclusion criteria included being aged 18+ years, living in a county with a Rural-Urban Continuum Code ≥ 6, living in a rural area for ≥10 years, and reporting eating ≥1 serving of meat per week. Participants in the first set of interviews were classified as having or not having a PBD based on their self-described eating patterns (assessed by two independent reviewers and determined through discussion). The second set of interviews (*n* = 28), conducted between May and June 2024, focused on plant-based eaters. For this, residents of Vermont (except for the most urban county, Chittenden) who were 18+ years old, lived in a rural area for ≥2 years, and self-identified as consuming a healthy PBD were recruited and interviewed. In line with recruitment criteria, all participants in the second set of interviews were considered to eat PBD. These sets of interviews offered complementary perspectives, with both interview guides addressing social influences on PBDs.

Data collection: All participants completed a sociodemographic survey before the interviews, which asked about age, gender, race, education, and income. Semi-structured interviews were conducted via phone or online video calls, allowing flexibility based on participants’ comfort and availability. Interviews lasted approximately 20 to 50 min. While interview questions for the first and second set of interviews differed (Supplementary Material S1) both were structured to explore the three main dimensions of TPB [12] as it relates to PBDs: attitudes, perceived behavioral control, and social norms. Both interview guides included a definition of a plant-based dietary pattern to support consistency in participant understanding across interviews (Supplementary Material S1) [13]. All interviews were audio-recorded with consent, transcribed, and checked for accuracy before being imported into NVivo 14 (QSR International, 2023) for coding and analysis.

Data analysis: The interview transcripts were repeatedly reviewed to gain an in-depth understanding of participants’ perspectives. For both interview sets, initial interviews were open coded by multiple team members who developed and refined codebooks based on emerging themes and the study’s theoretical framework. In addition to *a priori* codes, new codes were inductively developed to capture unexpected themes. Codes were clustered into higher-order themes consistent with TPB and iteratively refined to ensure accurate representation of participants’ experiences. Once the codebooks were finalized (Supplementary Material S2) and consistent coding was established, coding responsibilities were divided between team members. Following coding, code memos were drafted, discussed, and revised for each key theme.

For the present analysis, the first author reviewed code memos and re-read all interviews for text relevant to social dynamics to ensure complete knowledge of the data and identify sub-themes. These subthemes were noted and compared between the PBD and non-PBD study participants and across gender groups to identify gender-specific patterns in household dynamics and social influences.

## 3. Results

### 3.1. Study 1 (Survey)

Respondent characteristics: Table 2 presents respondent characteristics overall and by PBD adherence. Overall, approximately one-third (35.4%) reported following a PBD. The sample was diverse across age, gender, income, and education categories. Women were more likely than men to report following a PBD (*p* < 0.001). Compared to respondents aged 43–58 years and older, respondents aged 18–42 years were more likely to report following a PBD (*p* < 0.001). Additionally, respondents with postgraduate/professional degrees were more likely to report following a PBD than those with a high school degree or less (*p* = 0.004). Respondents following a PBD reported higher agreement that important others believed they should follow a PBD (*p* < 0.001).

**Figure 1:**
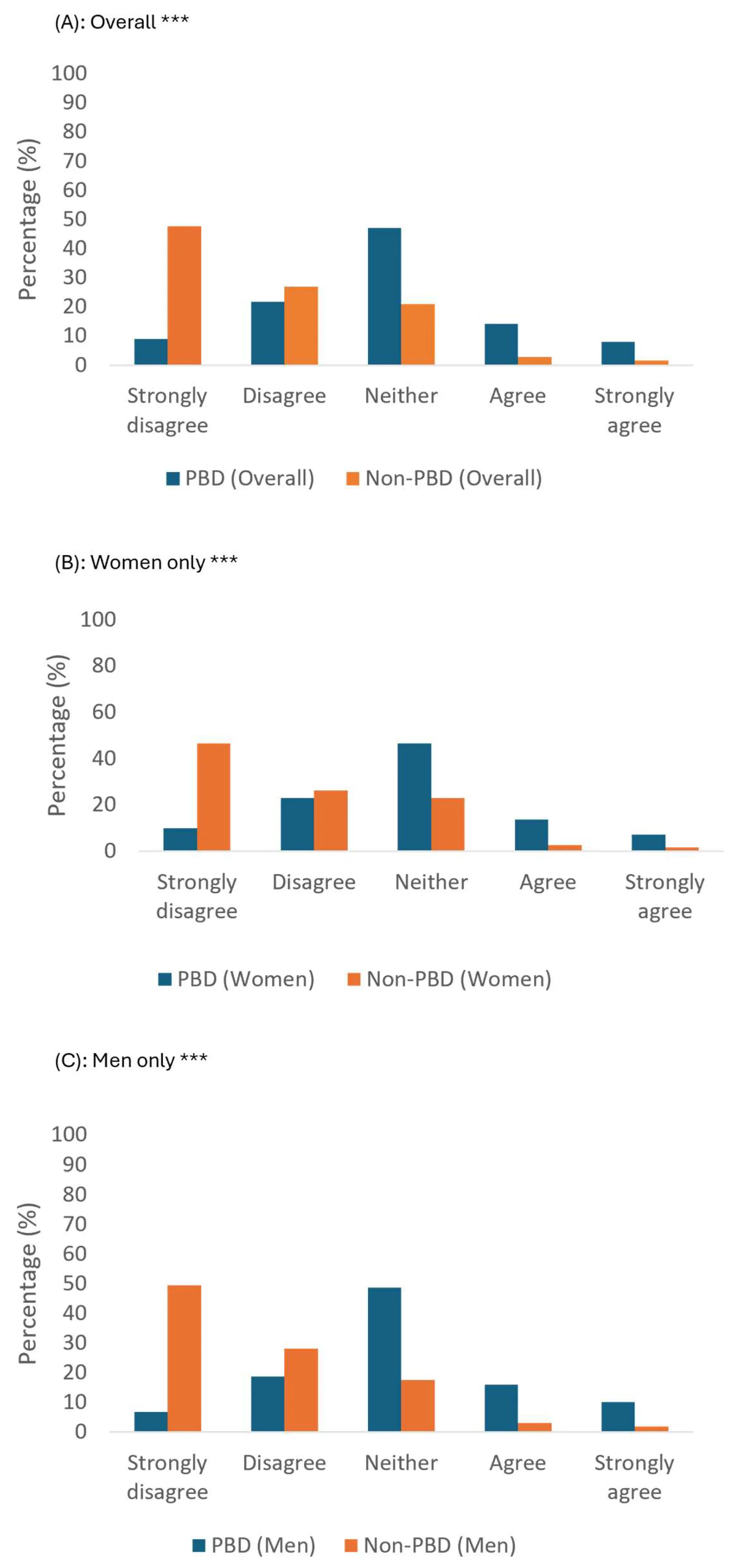
Distribution of agreement levels to “Most people who are important to me think I should follow PBD” among respondents stratified by PBD adherence: (**A**) all respondents, (**B**) women, and (**C**) men. Asterisks indicate statistically significant differences between PBD and non-PBD groups (*** *p* < 0.001).

**Figure 2:**
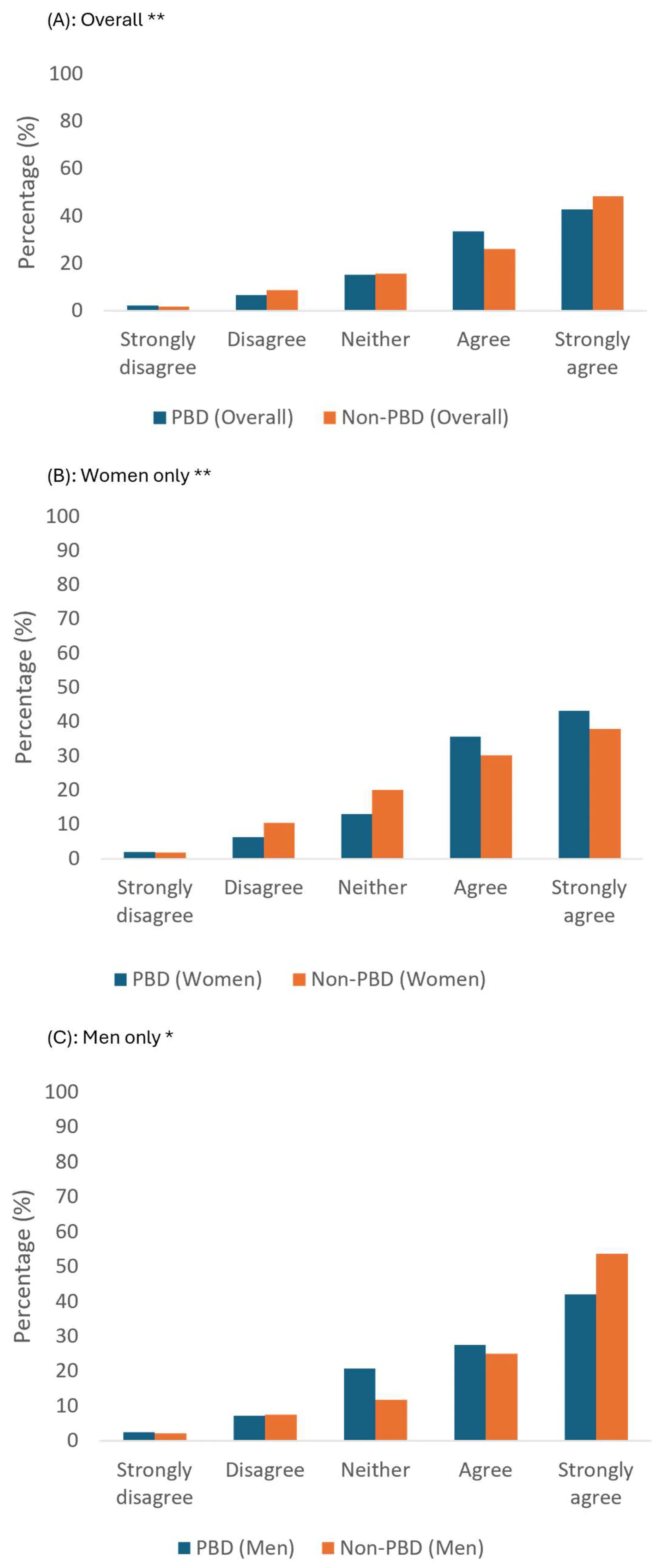
Distribution of agreement levels to “I feel social pressure to follow a plant-based diet” among respondents stratified by PBD adherence: (**A**) all respondents, (**B**) women, and (**C**) men. Asterisks indicate statistically significant differences between PBD and non-PBD groups (* *p* < 0.05, ** *p* < 0.01).

Figure 1 and Figure 2 present the social influence variables by PBD adherence, overall and stratified by gender. Respondents who adhered to a PBD reported higher perceived expectations from people important to them to follow a PBD compared with non-adherents (*p* < 0.001), a pattern that was consistent among both women and men. The distribution of perceived social pressure differed by PBD adherence overall (*p* = 0.003), among women (*p* = 0.001), and among men (*p* = 0.011).

Regression modeling: Respondents’ agreement that important people thought they should follow a PBD was associated with following a PBD overall (Wald χ^2^ (4) = 387.48, *p* < 0.001; Table 3). Šidák-adjusted pairwise comparisons indicated higher odds of reporting a PBD with increasing agreement, with significant differences between every response category except “agree” and “strongly agree” (*p* = 1.000). Respondents who agreed or strongly agreed had higher odds than those who neither agreed nor disagreed (OR (CI) = 2.16 (1.23–3.81), *p* = 0.001 and OR (CI) = 2.14 (1.04–4.41), *p* = 0.033, respectively); all remaining pairwise comparisons were significant at *p* < 0.001. However, the overall interaction with gender was not significant (Wald χ^2^(4) = 6.53, *p* = 0.163; Table 4).

Perceived social pressure was associated with PBD adherence overall (Wald χ^2^ (4) = 13.48, *p* = 0.009; Table 3). Šidák-adjusted pairwise comparisons showed that respondents who agreed had higher odds of reporting a PBD than those who strongly agreed (OR (CI) = 1.39 (1.03–1.88), *p* = 0.022) or disagreed (OR (CI) = 1.72 (1.02–2.93), *p* = 0.038). No other pairwise comparisons were significant. Moreover, this association differed by gender (Wald χ^2^ (4) = 18.08, *p* = 0.001; Table 4). In the interaction model, men who strongly agreed had lower odds of reporting a PBD than men who neither agreed nor disagreed (OR = 0.39, 95% CI: 0.18–0.84; adjusted *p* = 0.003). Among respondents who strongly agreed, women had higher odds than men (OR = 1.87, 95% CI: 1.14–3.05; adjusted *p* = 0.002).

### 3.2. Study 2 (Interviews)

Of the 55 adults who participated in semi-structured interviews, 34 followed a PBD (Table 5). Most were female (70.9%), midlife or older (72.7%), lived in Vermont (87.3%), had an associate or bachelor’s or postgraduate degree (70.9%), and lived in a household with an annual income less than $100,000 (78.2%). Participants described the powerful role of social relationships in shaping dietary behaviors, with emerging themes aligned with three realms of influence: immediate family and household dynamics, extended family and friends, and broader community norms. In the sections below, these themes are presented with illustrative quotes in the text. Further quotes and elaboration are presented in Supplementary Material S3.

Immediate Family: Most participants (*n* = 33) described ways in which their everyday family dynamics helped or hindered consumption of a PBD, with clearly gendered responses (Supplementary Material S3). Almost half (47%) of the PBD group (*n* = 16) described full household alignment with plant-based eating, which made meal planning simpler and reinforced their commitment. One participant recalled how she removed all animal products from their household diet and her husband just “went along with it”. Another described how she and her partner took years to bond over shared dietary values, years after he had originally “gone vegan for health reasons”. Some plant-based participants encouraged their less-plant-based partners to eat more PBD, especially if they were primary cooks in the household. For example, one female interview participant said “My partner…. eats mostly a PBD because I cook for him, but he doesn’t really have objections to it.” For others, their partner’s prior or greater commitment encouraged them. For instance, one woman reflected on how her husband’s preference for vegetables positively influenced her preference for vegetables. However, for nearly one-third of participants in the PBD group (*n* = 10; 29.4%), household members were not supportive of adopting a PBD, leading to practical challenges. One woman described how she cooked pasta but kept the meat “on the side” for her boyfriend. Another mentioned she still bought meat for her husband, even though she wouldn’t eat it herself. There was a clear pattern of gender differences related to family support. Within the PBD group, 60% of men (*n* = 6) explicitly described support from immediate family compared to 42% of women (*n* = 10). In contrast, among plant-based eaters, a lack of family support was described by 20% of men (*n* = 2) compared to 33% of women (*n* = 8).

In the non-PBD group, one third of participants (*n* = 7; 33.3%) noted their immediate families would not respond positively to plant-based meals. For instance, one woman said, “If there’s no meat, the kids ask, ‘Where’s the meat?’” A similar number (*n* = 9; 42.9%) thought their households would be open to PBD. For example, one woman described her husband as “totally on board with this [PBD], mainly for the environmental reasons”.

Extended Family: In both dietary pattern groups, participants shared how their food choices were shaped, to a lesser degree, by extended family (Supplementary Material S3). Extended family was discussed by about half of the PBD participants. Some (*n* = 9; 26.5%) reported that their extended family members were not supportive of their PBD, particularly in contexts where food traditions were strong or culturally significant. For example, one woman from a large Italian family said relatives would look at her and ask, “What are you going to eat?” Others (*n* = 9; 26.5%) described extended families who were already eating a PBD as supportive from the outset, or shared that they had become more open and accepting over time. For instance, one woman described how her relatives began trying and enjoying her plant-based dishes over the years and eventually became more supportive and curious about plant-based eating.

Approximately one-quarter of non-PBD participants (*n* = 6; 28.6%) discussed the perspectives of their extended family and friends, with equal numbers describing support for and resistance to plant-based eating.

Friends: Half of PBD participants (*n* = 17; 50%) described friends as a critical source of support. Several reflected on how having “like-minded” or “chosen” friends supported adherence to a PBD. Few PBD participants (*n* = 3; 8.8%) reported having friends who were unsupportive. In the non-PBD group, 6 (28.6%) described mixed reactions from friends, with some noting supportive vegetarian or vegan friends and others reporting friends who prefer meat-based diets.

Community Norms: Participants described a range of experiences related to broader community norms. In Vermont, one third of PBD participants (*n* = 11; 33.3%) spoke about the state’s deep-rooted hunting and dairy traditions as challenges to their dietary choices. One female PBD eater mentioned that “Vermont is very meat-based, hunting, dairy culture” oriented, and that “there’s a lot of stigma in general about people who would consider themselves plant-based”. While the same number (*n* = 11; 32.4%) in the PBD group pointed to Vermont’s progressive food culture as a source of support. Participants described Vermont as having abundant co-ops, farmers’ markets, and a shared respect for home-grown food. “Everyone’s got a garden,” one participant remarked, noting how her small town had a farm stand at the entrance and everyone shopped there. Patterns of reporting on this theme did not appear to differ across gender lines.

All participants from Texas, including one having PBD described their communities as unsupportive of PBDs. They emphasized that local food cultures in Texas are deeply rooted in meat production and consumption, with livelihoods tied to ranching and large-scale animal agriculture. For example, one participant noted that “everybody that I know eats meat on a daily basis,” highlighting how normalized meat consumption is within their social environment. These accounts illustrate participants’ perceived social environments and should not be interpreted as representative of rural Texans’ perspectives.

Among non-PBD participants from Vermont, seven described their communities as supportive of PBDs, often citing local food environments that emphasize fresh produce, farmers’ markets, and natural food stores. For instance, one participant described that many people in their community “follow it or believe that it’s the way to go”. Another highlighted the prevalence of home gardening and local farm stands, suggesting a culture that values plant-forward eating, even if not strictly plant-based. However, three non-PBD Vermont participants perceived less enthusiasm, pointing out that hunting traditions and access to locally sourced meat remain important aspects of food culture in the state.

## 4. Discussion

This study examines the role of social influences on adherence to PBDs among rural adults in the United States. In the context of strong meat-eating traditions, we found that attitudes and behaviors of family members have important influences on plant-based eating, with notable differences observed between men and women. This paper aimed to synthesize findings across quantitative and qualitative data sources to better understand how social dynamics shape PBDs in rural communities. The quantitative and qualitative findings converge on the idea that the approval of important others (injunctive norms) and generalized social pressure (descriptive norms) are both relevant to dietary behavior. Regression analyses confirmed a positive association between perceived expectations from important others and PBD adherence. Pairwise comparisons showed progressively higher odds of reporting a PBD across categories, with no significant difference between “agree” and “strongly agree.” Perceived social pressure showed a less consistent pattern: respondents who agreed had higher odds of reporting a PBD than those who disagreed or strongly agreed, and the association varied by gender. Among respondents who strongly agreed that they felt social pressure to follow a PBD, women had higher odds of reporting a PBD than men. Our findings highlight the need for public health and clinical interventions in rural communities to consider the ways in which close relationships can be leveraged to support healthier and potentially more environmentally sustainable eating patterns, particularly given the high rates of cardiometabolic diseases in the U.S. and the known association to diet as a key driver. Although income was not significantly associated with PBD adherence, economic constraints may still influence dietary quality among those following a PBD [14].

Prior research points to the importance of household dynamics and shared routines among household members in shaping dietary decisions [7,15]. A study of meat eating among rural residents in rural central New York, found family expectations to consume meat were strongly correlated with meat consumption, but other measures of social norms (such as friends’ and neighbors’ norm) were not [9]. Similarly, in the current study, dietary alignment and support from household members emerged as a particularly impactful facilitator or hindrance to plant-based eating. Shared cooking, similar values, and encouragement from partners made it easier to adopt and maintain a PBD. In contrast, difficulties emerged when family members did not support plant-based eating, requiring compromise or separate meal preparation. Research on gendered foodwork shows how unequal responsibilities shape dietary choices [16]. Food agency involves the ability to achieve dietary goals within social and practical constraints, as well as cooking skills [17]. Shared meals within households serve functions that extend beyond basic nutrition. These moments are important for social connection and emotional bonding, which may lead family members to establish a dietary routine to improve these relationships [8]. Support from family members can facilitate dietary goals and commitments for plant-based eaters, while fractious reactions may have a negative impact [8]. Prior qualitative research with self-identified vegetarian Americans found that family members may undermine PBD efforts through actions such as minimizing the decision to eat a PBD as a short-term trend, questioning the eater’s intentions, and framing eating a PBD as a threat to family norms [18]. These themes also emerged in the present study. Many participants, especially women, emphasized how household alignment and family support either enabled or hindered PBD adoption. In our qualitative sample, a larger proportion of men than women described family support for plant-based dietary pattern. One possible explanation involves gendered divisions in household cooking. Prior research indicates that women more frequently prepare meals [19]; individuals less involved in cooking may depend more on household accommodation of their dietary preferences. Family support may therefore provide both emotional encouragement and practical access to plant-based meals. These interpretations draw on our multimethod findings and prior literature but remain hypotheses. Longitudinal research could clarify how social support changes during the adoption and maintenance of a PBD. Interventions that build food agency and cooking skills among all household members may help empower those who do not typically prepare food to make meals that align with their own goals.

The lower prevalence of PBDs among men compared to women in the quantitative analysis may be partially explained by the symbolic role of meat in reinforcing masculinity [10,20,21,22,23]. Studies have consistently shown that men who strongly identify with meat consumption perceive PBDs as threatening to their gender identity [10,20]. This may help explain the association between perceived social pressure (descriptive norms) and its gender interaction. Among men, strongly agreeing that they experienced social pressure was associated with lower odds of reporting a PBD than a neutral response; among respondents who strongly agreed, women had higher odds than men. However, the survey did not assess whether masculinity or attitudes toward meat explained the interaction. No within-women pairwise comparison remained significant after adjustment, which should not be interpreted as evidence that social pressure is unrelated to PBD adherence among women.

Looking at social support from extended family members and friends, participants reported a wide range of experiences. While some PBD participants noted encouragement or acceptance, others mentioned resistance rooted in tradition or inherited culture about meat. For the PBD group, friends were more supportive, especially when they shared ethical or environmental values, acting as facilitators in reinforcing plant-based behaviors. Non-PBD participants more often described mixed attitudes from their social circles.

In the rural communities where interview participants in this study lived, cultural norms around raising, hunting, and eating animals shaped views on the value of meat in the diet. For example, deep ties to ranching and hunting among some residents constrained acceptance of PBDs, while local cultures believed to be more accepting of plant-based eating supported PBD adoption. The associations observed in our models highlight the relevance of subjective norms within the TPB [12]. These findings suggest that perceived expectations and felt social pressure may relate differently to PBD. In livestock-producing communities, programs could address potential cultural and economic concerns by involving local producers and emphasizing plant foods within familiar meals that include some animal products.

Taken together, the findings in both complementary data sets suggest that successful promotion of healthy PBDs must attend to multi-level social dynamics in rural communities. At the micro level, family support and household practices directly shape dietary feasibility, particularly for men less engaged in cooking. At the macro level, subjective norms from close social circles may have the strongest influence. Furthermore, cultural identity and masculinity norms in rural communities remain substantial barriers to male adoption of PBDs. These findings suggest the need for targeted interventions, for example agricultural extension initiatives, cooking courses, and culinary medicine workshops that explicitly consider household dynamics and discussions around food.

Strengths of this paper include using data from two complementary data sets and a large sample size for the quantitative study, which allowed for both statistical modeling and in-depth exploration of lived experiences. Limitations include the cross-sectional nature of the quantitative data and the reliance on self-reported dietary pattern adherence. Although participants across both datasets were provided with a standardized definition of a plant-based dietary pattern, adherence was assessed through self-identification rather than dietary intake assessment (e.g., 24-h dietary recall). Therefore, there was likely variation in how participants interpreted or applied the definition to their own eating behaviors. Additional research is needed to validate the dietary pattern question used in this study. Social influences were assessed using two agreement items, each analyzed separately as an observed item. These measures do not constitute a comprehensive assessment of social norms. Moreover, high proportion of women and postsecondary degree holders limit the sample’s representativeness of rural U.S. adults. Given that research suggests that PBDs rich in fruits, vegetables, whole grains, and legumes are associated with positive health outcomes and that PBDs high in refined grains and added sugars are detrimental, future studies should include additional measures to characterize diet quality. Future research should also include additional measures of both injunctive and descriptive norms to more fully explore the mechanisms through which social dynamics work to shape PBDs. Additional work is also needed to elaborate the experiences of adhering to a PBD in rural versus nonrural areas and to understand how gender interacts with social factors to shape dietary patterns, especially in communities where traditional gender roles continue to dominate.

## 5. Conclusions

This research shows that social influences play an important role in shaping adherence to PBDs among rural adults with potential implications for resilient and sustainable food systems. Across both quantitative and qualitative analyses, the support of important others—especially those living in the same home—emerged as the most consistent predictor of adherence to PBDs. The findings highlight that family dynamics can either facilitate or constrain plant-based eating, with shared cooking, aligned values, and supportive partners enabling adherence, while resistance within the home creates practical and emotional barriers. Notably, men had lower rates of PBD adherence, and the association between perceived social pressure and PBD adherence differed by gender. These patterns may reflect existing gendered divisions in food preparation, whereby men depend more heavily on family support for dietary change. Interventions to address dietary change among rural residents will likely encounter and should account for sociocultural and contextual considerations as well as gendered food identities.

## Figures and Tables

**Table 1:** Survey variables used to examine relationships between social norms and adherence to PBD.

Variable Type	Variable	Values
Dependent	Plant-based dietary pattern	Yes, No
Categorical	Age group	18–42, 43–58, 59+ years
Categorical	Gender	Woman, Man
Categorical	Household income (2022, before taxes)	≤$24,999, $25,000–74,999, $75,000–99,999, ≥$100,000
Categorical	Education level	High School degree or less, Some college, Associate or Bachelor, Postgraduate/Professional
Categorical	I feel social pressure to follow a PBD	Strongly disagree (1), Disagree (2), Neither agree, nor disagree (3), Agree (4), Strongly agree (5)
Categorical	Most people who are important to me think I should follow a PBD	Strongly disagree (1), Disagree (2), Neither agree, nor disagree (3), Agree (4), Strongly agree (5)

**Table 2:** Sociodemographic characteristics and social norms measures among study respondents, overall and stratified by PBD adherence.

Characteristic	Total Respondents ^‡^	PBD *n* (%)	No PBD *n* (%)	*p*-Value
PBD adherence	2210	782 (35.4%)	1428 (64.6%)	
Age group				<0.001
18–42 years old	895	378 ^b^ (48.3%)	517 ^a^ (36.2%)	
43–58 years old	723	251 ^a^ (32.1%)	472 ^a^ (33.1%)	
59 years and older	592	153 ^b^ (19.6%)	439 ^a^ (30.7%)	
Gender				<0.001
Woman	1457	574 ^b^ (73.4%)	883 ^a^ (61.8%)	
Man	753	208 ^b^ (26.6%)	545 ^a^ (38.2%)	
Annual household income				0.222
≤$24,999	368	134 ^a^ (17.1%)	234 ^a^ (16.4%)	
$25,000–74,999	929	308 ^a^ (39.4%)	621 ^a^ (43.5%)	
$75,000–99,999	388	138 ^a^ (17.6%)	250 ^a^ (17.5%)	
≥$100,000	525	202 ^a^ (25.8%)	323 ^a^ (22.6%)	
Education level				0.004
High school degree or less	205	58 ^b^ (7.4%)	147 ^a^ (10.3%)	
Some college (No degree)	444	150 ^a^ (19.2%)	294 ^a^ (20.6%)	
Associate or bachelor’s degree	986	338 ^a^ (43.2%)	648 ^a^ (45.4%)	
Postgrad/professional degree	575	236 ^b^ (30.2%)	339 ^a^ (23.7%)	
I feel social pressure to follow PBD	2186	775	1411	0.003
Strongly disagree	40 (1.8)	16 ^a^ (2.1)	24 ^a^ (1.7)	
Disagree	172 (7.9)	51 ^a^ (6.6)	121 ^a^ (8.6)	
Neither agree nor disagree	335 (15.3)	117 ^a^ (15.1)	218 ^a^ (15.5)	
Agree	626 (28.6)	259 ^b^ (33.4)	367 ^a^ (26.0)	
Strongly agree	1013 (46.3)	332 ^b^ (42.8)	681 ^a^ (48.3)	
Most people who are important to me think I should follow PBD	2209	781	1428	<0.001
Strongly disagree	750 (34.0)	70 ^b^ (9.0)	680 ^a^ (47.6)	
Disagree	554 (25.1)	170 ^b^ (21.8)	384 ^a^ (26.9)	
Neither agree nor disagree	667 (30.2)	368 ^b^ (47.1)	299 ^a^ (20.9)	
Agree	152 (6.9)	111 ^b^ (14.2)	41 ^a^ (2.9)	
Strongly agree	86 (3.9)	62 ^b^ (7.9)	24 ^a^ (1.7)	

^‡^ The sample size includes respondents with no missing values in all variables for at least one model. Column proportions with different superscripts differ significantly based on Bonferroni-adjusted pairwise comparisons (*p* < 0.05).

**Table 3:** Overall associations with PBD in two adjusted logistic regression models.

Characteristic	“Perceived Influence from Important People” Model: Wald χ^2^ (df)	*p*-Value	“Social Pressure” Model: Wald χ^2^ (df)	*p*-Value
Age	36.970 (2)	<0.001	31.319 (2)	<0.001
Education	5.899 (3)	0.117	10.907 (3)	0.012
Income	3.587 (3)	0.310	4.660 (3)	0.198
Gender	11.295 (1)	<0.001	13.420 (1)	<0.001
Most people who are important to me think I should follow PBD	387.482 (4)	<0.001	–	–
I feel social pressure to follow PBD	–	–	13.483 (4)	0.009

Dependent variable: PBD. Perceived influence from important people model: *n* = 2209; (Hosmer–Lemeshow χ^2^ = 7.80, *p* = 0.453); social-pressure model: *n* = 2186; (Hosmer–Lemeshow χ^2^ = 7.85, *p* = 0.448); Dashes indicate that the predictor was not included in that model. Each test adjusts for: age, education, income, gender.

**Table 4:** Overall associations of gender interactions with social norm measures in adjusted logistic regression models of PBD adherence.

Characteristic or Interaction Term	“Perceived Influence from Important People” Model: Wald χ^2^ (df)	*p*-Value	“Social Pressure” Model: Wald χ^2^ (df)	*p*-Value
Gender × perceived influence from important people	6.533 (4)	0.163	–	–
Gender × social pressure	–	–	18.084 (4)	0.001

Dependent variable: PBD. Perceived influence from important people model: *n* = 2209; (Hosmer–Lemeshow χ^2^ = 5.69, *p* = 0.682); social-pressure model: *n* = 2186; (Hosmer–Lemeshow χ^2^ = 7.77, *p* = 0.456). Dashes indicate that the predictor was not included in that model. Each Wald test adjusts for: age, education, income, gender, the corresponding social norm, and its gender interaction. Main effects among men and gender are evaluated at “strongly disagree.”

**Table 5:** Participants’ demographics in the qualitative study.

Category	Variable	PBD (*n* = 34)	Non-PBD (*n* = 21)	Total (*n* = 55)
Gender	Female	24 (61.5%)	15 (38.5%)	39
	Male	10 (62.5%)	6 (37.5%)	16
State	Vermont	33 (68.7%)	15 (31.3%)	48
	Texas	1 (14.3%)	6 (85.7%)	7
Age group	18–42 years old	10 (66.7%)	5 (33.3%)	15
	43–58 years old	7 (58.3%)	5 (41.7%)	12
	59 years or more	17 (60.7%)	11 (39.3%)	28
Education	High school or less	0 (0.0%)	2 (100.0%)	2
	Some college (No degree)	4 (44.4%)	5 (55.6%)	9
	Associate or bachelor’s degree	15 (60%)	10 (40%)	25
	Postgraduate/Professional degree	14 (82.4%)	3 (17.6%)	17
	Not reported	1 (50%)	1 (50%)	2
Annual household income	≤$24,999	8 (66.7%)	4 (33.3%)	12
	$25,000–74.999	10 (47.6%)	11 (52.4%)	21
	$75,000–99.999	9 (90.0%)	1 (10.0%)	10
	≥$100,000	7 (63.6%)	4 (36.4%)	11
	Not reported	—	1 (100.0%)	1

## Data Availability

The data presented in this study are available on request from the corresponding author due to privacy restrictions.

## Supplementary Materials

The following supporting information can be downloaded at https://www.mdpi.com/article/10.3390/nu18203346/s1. Supplementary Material S1: Interview guides used for the two sets of interviews. Supplementary Material S2: Coding list and description of codes. Supplementary Material S3: Themes and illustrative quotes from the qualitative study.
