# Supplementary material for: Social Dynamics Are Associated with Plant-Based Dietary Consumption Patterns Among Rural Adults in the U.S
Source: Nutrients. 2026 Oct 13;18(20):3346. doi: 10.3390/nu18203346 (PMC13708334; doi:10.3390/nu18203346)
Supplement: Supplementary file 1 [file nutrients-18-03346-s001.zip › Supplementary material S1.pdf]

Supplementary material S1
Interview guides used for the two sets of interviews

Interview questions guide for the first recruitment	
Overarching Question	Interview Question
What is the understanding of plant-based diets?	1. Can you tell me what is a typical meal your family eats for dinner?<br>2. When you think of good or satisfying food, what comes to mind?<br>3. If you were to think of unpleasant or unsatisfying food, what comes to mind?<br>4. How would you describe the role of meat in how you eat?<br>5. When considering your general health, how important do you think it is to eat meat?<br>6. Have you ever heard the term “plant-based diet”? what do you think it means?<br>7. What do you see as advantages of eating a plant-based diet?<br>8. What do you see as disadvantages of eating a plant-based diet, if any?<br>9. What positive feelings do you associate with eating a plant-based diet, if any?<br>10. What negative feelings do you associate with eating a plant-based diet?
What is the role of upbringing and social support to adhere to a plant-based diet?	11. What role did meat play in your diet when you were growing up?<br>12. How do you believe your family views plant-based diets?<br>13. How do you think that your extended family would view plant-based diets?<br>14. How do you believe your community more broadly views plant-based diets?
What is the perceived behavioral control related to a plant-based diet?	15. Do you think that eating a plant-based diet would be easy or challenging, and can you explain?<br>16. If you wanted to eat a plant-based diet, what do you think would make it easier for you?<br>17. If you want to eat a plant-based diet again, what would make that more difficult?

Interview questions guide for the second recruitment	
Overarching Question	Interview Question
Opening Questions and showing the diagram of a plant-based diet from the EAT-Lancet Commission on Food, Planet, Health (https://eatforum.org/eat-lancet-commission/the-	1. Can you tell me about what you ate for dinner last night and whether this is a “typical” meal for you?<br>2. Overall, how would you describe the way that you eat?<br>3. During this interview we are going to use the term ‘plant-based diet’. When we use the term diet, we are not referring to calorie restriction related to weight loss. But we are talking about a pattern of eating. We define a plant-based diet as a dietary pattern dominated by

planetary-health-diet-and-you/)	fresh or minimally processed plant foods and decreased consumption of meat, eggs and dairy products. Compared to meat-centered diets, plant-based diets involve increased consumption of a variety of grains, fruits, vegetables, beans, nuts and seeds. So it does not necessarily mean a vegetarian diet and can contain modest amounts of fish, meat, and dairy. [25]<br>4. Based on the way that you answered questions on our survey, we have categorized the way you eat as ‘plant-based’
Personal perspectives and experiences of eating plant-based	5. I’m curious to learn more about why you eat a plant-based dietary pattern. What are some of the reasons that you do it?<br>6. What was your experience starting a plant-based dietary pattern?<br>7. On a 10-point scale from ‘very easy’ to ‘very difficult’, how would you categorize your personal experience eating a plant-based diet in rural?
Outside perceptions of plant-based diet	8. What do your friends and family think about the way that you eat?<br>9. What do you think other people in your community think about plant-based diets?
Community recommendations	10. If you were to give tips to a friend or a neighbor about how to eat a plant-based diet, what would you say?<br>11. If you could make a change in your community that would make it easier for other people to eat plant-based, what would it be?
Questions about nutrients of concern	12. Do you have any concerns about the healthfulness of a plant-based diet?<br>13. What do you think about when choosing plant-based protein foods?<br>14. Do you seek out any specific foods or food combinations to fill in possible gaps in your diet from avoiding/eating very little animal products?<br>15. Specific questions based on responses to the survey such as: I see that you take Vitamin B12; can you talk about your decision to take this supplement? Or I see that you eat nutritional yeast more than once a week; can you talk about the reasons why?
Closing questions	16. Is there anything else about eating a plant-based diet in your community that you would like to share?<br>17. Is there anything else about diet planning that we haven’t talked about that you would like to mention?
