# Supplementary material for: Social Dynamics Are Associated with Plant-Based Dietary Consumption Patterns Among Rural Adults in the U.S
Source: Nutrients. 2026 Oct 13;18(20):3346. doi: 10.3390/nu18203346 (PMC13708334; doi:10.3390/nu18203346)
Supplement: Supplementary file 1 [file nutrients-18-03346-s001.zip › Supplementary material S2.pdf]

Supplementary material S2
Coding list and description of codes

Code Name	Description
**Social support (Immediate Family)**	To support or not support plant-based choices within the household.
**Social support (Extended Family)**	To support or not support plant-based choices in the extended family.
**Social support (Friends)**	To support or not support plant-based choices in friends.
**Community norm**	To support or not support plant-based choices in the broader community
