# Supplementary material for: Social Dynamics Are Associated with Plant-Based Dietary Consumption Patterns Among Rural Adults in the U.S
Source: Nutrients. 2026 Oct 13;18(20):3346. doi: 10.3390/nu18203346 (PMC13708334; doi:10.3390/nu18203346)
Supplement: Supplementary file 1 [file nutrients-18-03346-s001.zip › Supplementary material S3.pdf]

Supplementary material S3

Themes and illustrative quotes from the qualitative study.

Themes	Subthemes relevant to Plant-based group	Example	Subthemes relevant to the non-plant-based group	Example
Immediate family	Household alignment with PBD	I made that change probably about six years ago, completely removing all animal products from our diet. And fortunately, my husband was happy to go along with that. (P2VT07, PBD)<br><br>... My partner went vegan for health reasons originally first though now we share a lot of values with the animal rights stuff . (P2VT24, PBD).	Not positive towards PBD	[My husband] doesn't influence my choices at all, but I can't cook anything unless it's meat based for him and the kids. Because if I do, it's like, "Where's meat? Where's the meat?" And the kids feed off that, they idolize him. So of course, whatever he says is God's word, which I get. (P1VT21, non-PBD)
	They encouraged their less-plant-based partners to eat a more PBD	I live with my wife and my two teenage sons and they aren't as into it [PBD]. And that's fine, I'm the cook so I end up controlling the dinner most of the time. But we kind of have a [policy]: you're on your own for breakfast and lunch policy in our house. And then, I cook dinner. So that way, people feel like they can get their own needs met. But I can	Their family would be positive to have a PBD	[Eating a PBD] would be 100% acceptable [to] my husband... Even though we enjoy meat, once a week [eating a PBD] would be fine for him. (P1TX07, non-PBD)<br><br>My wife does not eat red meat. I don't know if she likes it. I think she stays away from it for health. (P1VT15, non-PBD)

	actually still eat the way that I want to. That seems like a good compromise. (P2VT14, PBD)<br><br>He (partner) feels sometimes his body does a little better when he eats animal products, so occasionally he will crave a burger and get a burger, eat some chicken. But for the most part he never really objects to the way I cook and doesn't feel like unsatisfied with the meals. (P2VT16, PBD)	
Their partner's prior or greater commitment encouraged them in return	My husband doesn't eat much chicken. He doesn't eat the chicken usually when I cook it. So, because he eats a lot of vegetables, he [eats] PBD, then I do that too. (P2VT04, PBD)	
Immediate families are not supportive of PBD	I make separate meals when I'm eating with my [boyfriend]. Or I'll try to incorporate some of the same stuff like just eating pasta, keeping the chicken separate from the pasta. (P2VT13, PBD)<br><br>I live with my wife and my two teenage sons and they aren't as into	

		it (PBD). (P2VT14, PBD)<br><br>I'd make a wonderful dish. He (husband) would cut up the sausage and put it on top of it. (P2VT05, PBD)		
Extended family	Not supportive	My family haven't always been super supportive. And, we present ourselves as a challenge to our families when we're not eating certain things, even without the red meat -- that was perceived as a challenge for our families. What are they going to feed us? (P2VT01, PBD)<br><br>[I have] a big Italian family and everybody's like, "what are you gonna eat? What are we gonna feed you?" They got better when I said I wouldn't do like the vegan stuff anymore. They didn't give me necessarily a hard time. They just were confused. (P2VT13, PBD)	A mix of both positive and negative attitudes	Even in my extended family, my brother and his son, my brother's gluten free and then my nephew is a type one diabetic. So even at our big family gatherings, we've had to change, and we have a lot of vegan folks in our family too. So, we definitely have a lot of different more options when we're having a big meal for our family. (P1VT08, non-PBD)

	Always supportive or have become more accepting over time	In terms of like my larger network like family, they always find it really interesting and over the years my family's gotten more adventurous in trying things that I'm making and really enjoying them. (P2VT16, PBD)<br><br>We do have family that eat pretty much plant based. My wife's mother and her husband eat primarily plant based. They are curious about it, and they've always been pretty health-conscious people. (P2VT25, PBD)		
Friends	Supportive	Back in 1975... I think having my friends around me that were all doing it [eating plant-based], and they were good chefs. So, they were coming up in some nice vegetarian food too, stuff like that. (P2VT18, PBD)<br><br>My friends, like chosen friends, are probably similar to me, similar to my feelings, so favorable of a PBD. (P1VT02, PBD)	A mix of both positive and negative attitudes	I think a lot of my friends eat a lot of protein, a lot of meat. They think they need to have it. So, some friends are vegetarian, but a lot of them do the same thing as I do and eat a lot of local meat. Maybe more so than I do. (P1VT17, non-PBD)
	Not supportive	I actually have some friends who are pretty extreme meat eaters. (P1VT09, PBD)		

Broader community	Meat is culturally important in Vermont	I think [livestock] would be part of it. I don't hunt, but I know that's somewhat big in Vermont. I'd guess that most people are pretty much the meat and potatoes kind of people. (P2VT28, PBD)<br><br>A lot of people [from Vermont] hunt for their food…. I feel like hunters respect the land for the most part. And we have a lot of farms around here that raise [livestock]... my principal's husband has a farm where he raises grass fed beef. (P2VT10, PBD)	Meat is culturally important in Texas	We've got an awful lot of people around here that their income is dependent on meat production. A lot of cattle are in the area, a lot of farmers around, there are a lot of ranchers... I know one girl that probably is a vegetarian, I guess. Just everybody that I know eats meat on a daily basis. (P1TX01, Non-PBD)<br><br>In our community they're so like into hunting, and their meals are mostly based around meat. (P1TX04, Non_PBD)
	Vermont communities would be positive towards PBD	I have never heard anything negative in Vermont [about a PBD]. My experience with the people that I have interacted with in Vermont has been very supportive of healthy growing and healthy eating. I don't think I've ever had a negative experience here in Vermont. (P2VT09, PBD)	Vermont communities would be positive towards PBD	A lot of people, at least who I know, follow it (a PBD) or believe that it's the way to go. And there is that huge natural food store and there's the farmer's market. I think people view it positively in my community. (P1VT06, Non-PBD)<br><br>I think it's (a PBD) viewed rather positively, but I think a lot of people are in the same boat as I

			am. You eat meat in moderation, because it is expensive. (P1VT10, Non-PBD)
		Vermont communities would not be positive towards PBD	In Vermont there are probably a higher percentage of meat eaters, than maybe some other places. Hunters... have their freezers stocked full of meat that they've caught. (P1VT15, non-PBD)
